# Quality of life in patients with food allergy

**DOI:** 10.1186/s12948-016-0041-4

**Published:** 2016-02-17

**Authors:** Darío Antolín-Amérigo, Luis Manso, Marco Caminati, Belén de la Hoz Caballer, Inmaculada Cerecedo, Alfonso Muriel, Mercedes Rodríguez-Rodríguez, José Barbarroja-Escudero, María José Sánchez-González, Beatriz Huertas-Barbudo, Melchor Alvarez-Mon

**Affiliations:** Servicio de Enfermedades del Sistema Inmune-Alergia, Hospital Universitario Príncipe de Asturias. Departamento de Medicina y Especialidades Médicas, Universidad de Alcalá, Carretera de Alcalá-Meco s/n, 28085 Alcalá de Henares, Madrid, Spain; Hospital del Sureste. Arganda del Rey, Unidad de Alergia, Madrid, Spain; Allergy Unit, Verona University and General Hospital, Verona, Italy; Servicio de Alergia, Hospital Universitario Ramón y Cajal, IRYCIS, Madrid, Spain; Servicio de Alergia, Hospital Universitario Clínico San Carlos, Madrid, Spain; Unidad de Bioestadística Clínica, Hospital Universitario Ramón y Cajal, IRYCIS, Madrid, Spain

**Keywords:** Quality of life, Food allergy, Questionnaire, Specific questionnaire, Health-related quality of life (HRQL), Anaphylaxis

## Abstract

Food allergy has increased in developed countries and can have a dramatic effect on quality of life, so as to provoke fatal reactions. We aimed to outline the socioeconomic impact that food allergy exerts in this kind of patients by performing a complete review of the literature and also describing the factors that may influence, to a greater extent, the quality of life of patients with food allergy and analyzing the different questionnaires available. Hitherto, strict avoidance of the culprit food(s) and use of emergency medications are the pillars to manage this condition. Promising approaches such as specific oral or epicutaneous immunotherapy and the use of monoclonal antibodies are progressively being investigated worldwide. However, even that an increasing number of centers fulfill those approaches, they are not fully implemented enough in clinical practice. The mean annual cost of health care has been estimated in international dollars (I$) 2016 for food-allergic adults and I$1089 for controls, a difference of I$927 (95 % confidence interval I$324–I$1530). A similar result was found for adults in each country, and for children, and interestingly, it was not sensitive to baseline demographic differences. Cost was significantly related to severity of illness in cases in nine countries. The constant threat of exposure, need for vigilance and expectation of outcome can have a tremendous impact on quality of life. Several studies have analyzed the impact of food allergy on health-related quality of life (HRQL) in adults and children in different countries. There have been described different factors that could modify HRQL in food allergic patients, the most important of them are perceived disease severity, age of the patient, peanut or soy allergy, country of origin and having allergy to two or more foods. Over the last few years, several different specific Quality of Life questionnaires for food allergic patients have been developed and translated to different languages and cultures. It is important to perform lingual and cultural translations of existent questionnaires in order to ensure its suitability in a specific region or country with its own socioeconomic reality and culture. Tools aimed at assessing the impact of food allergy on HRQL should be always part of the diagnostic work up, in order to provide a complete basal assessment, to highlight target of intervention as well as to evaluate the effectiveness of interventions designed to cure food allergy. HRQL may be the only meaningful outcome measure available for food allergy measuring this continuous burden.

## Background

Food allergy (FA) has increased in developed countries and can have a dramatic effect on quality of life, so as to provoke fatal reactions [1–4]. We aimed to outline the socioeconomic impact that food allergy exerts in this kind of patients, by performing a complete review of the literature and also describing the factors that may influence, to a greater extent, the quality of life (QoL) of patients with food allergy. Moreover, the impairment in QoL may differ depending on the age, and as several specific questionnaires have been developed, we sought to describe into detail the different questionnaires available (Tables 1, 2, 3). Besides, as the terminology used with regards QoL is concrete and presumably complex, we wanted to clarify it, providing succinct definitions, for the sake of clarity (Table 4).

**Table 1:** Children/adolescents food allergy specific QoL questionnaires

Questionnaire	#Items	Domains/covered issues	Age	Completed by	Result	Reliability	Validity	Patients included in development	References
Food allergy quality of life-parental burden (FAQL-PB)	17	Family, school and social events, time employed to prepare foods, physical and mental state	0–17	Parents	parents whose children had multiple (>2) food allergies were more affected than parents whose children had fewer allergies	Internal consistency (test–retest)	Internal: inter-item correlations; external: criterion validity, construct, content	Yes	Cohen et al., USA [26]
Food allergy impact scale (FAIS)	32	Family and social events, field trips, parties, sleepovers and playing at friends’ houses	0–18	Parents	Daily family life (Meal preparation and family social activities)	Internal consistency (test–retest)	Internal: not proven; external: content, face validity	Yes	Bollinger et al., USA [55]
Food allergy parent questionnaire (FAPQ)	18	Parental anxiety/distress, psychosocial impact of allergies, parental coping/competence, and family support	0–18	Parents	Greater number of food allergies, positive history of anaphylaxis: higher scores on the anxiety/distress and psychosocial impact subscales. Internal consistency good for the anxiety/distress and psychosocial impact subscales	Internal consistency (test–retest)	Internal: factor analysis; external: face-validity, content	No	LeBovidge et al., USA [56]
Child health questionnaire parental form-28 (CHQ-PF 28)	28	Issues related to children, parents and family	9	Parents	Lower scores for physical functioning and role/social limitations	Not proven	Not proven	Yes	Östblom et al., Sweden [57]
Food allergy self-efficacy scale for parents (FASE-P)	21	Managing Social activities precaution and prevention. Allergic treatment food allergen identification seeking information about food allergy	0–18	Parents	Poorer self-efficacy was related to egg and milk allergy; self-efficacy was not related to severity of allergy	Internal consistency	External: discriminative, face-validity, construct, convergent	Yes	Knibb et al., UK [22]
Pediatric allergic disease quality of life questionnaire (PADQLQ)	26	Practical problems, symptoms, emotional problems	6–16	Children	A potentially useful outcome measure in the evaluation of systemic treatments in children with multisystem allergic disease	Internal consistency	Internal: inter item-correlations; external: construct, longitudinal	Yes	Roberts et al., UK [58]
Food allergy quality of life questionnaire-parent form (FAQLQ-PF)	30	Emotional impact; food-related anxiety; dietary and social restrictions	0–12	Parents	Domains and total score improved significantly at pos-challenge time-points for pre-challenge and post-challenge. Poorer quality of life at baseline increased the odds by over 2.0 of no improvement in HRQL scores 6-month time-point	Internal consistency (test–retest)	Internal: inter-item correlations, factor analysis, ceiling/floor effect; external: face-validity, content, convergent/discriminative, construct	Yes	DunnGalvin et al., Ireland [21]
Food allergy quality of life questionnaire-child form (FAQLQ-CF)	24	Allergen avoidance and dietary restrictions; emotional impact; risk of accidental exposure;	8–12	Children	Discriminated between children who differed in number of food allergies (>2 food allergies) vs. < or = 2 food allergies	Internal consistency (test–retest)	Internal: inter-item correlations; external: face-validity, content, convergent/discriminative, construct	Yes	Flokstra-de Blok et al., The Netherlands [24]
Food allergy quality of life questionnaire-teenager form (FAQLQ-TF)	23	Allergen avoidance and dietary restrictions; emotional impact; risk of accidental exposure;	13–17	Children	Discriminated between children who differed in number of food allergies (>2 food allergies vs. < or = 2 food allergies)	Internal consistency (test–retest)	Internal: inter-item correlations; external: face-validity, content, convergent/discriminative, construct	Yes	Flokstra-Blok et al., The Netherlands [23]
Food allergy quality of life assessment tool for adolescents (FAQL-teen)	17	Impact of food allergy-related limitations, perception of food allergy as a burden; fear for allergic reactions; disappointment for carrying the adrenaline auto-injector	13–19	Children	Areas most troubling included limitations on social activities, not being able to eat what others were eating, and limited choice of restaurants	Internal consistency	External: face-validity, discriminative, Cross-sectional construct validity	Yes	Resnick et al., USA [39]
You and your food allergy	34	Social well-being and independence, support, day-to-day activities, family relations and emotional well-being	13–18	Children	Discriminates by disease severity	Internal consistency (test–retest)	Internal: inter-item correlations; external: convergent/discriminative, construct	Yes	MacKenzie et al., UK [40]

**Table 2:** Adult food allergy specific questionnaires

Questionnaire	#Items	Domains	Age	Completed by	Result	Reliability	Validity	Patients included in development	References
Food allergy quality of life questionnaire-adult form (FAQLQ-AF)	29	Allergen avoidance and dietary restrictions; emotional impact; risk of accidental exposure; Food allergy related health	≥18	Adults	Discriminated between patients who differ in severity of symptoms (anaphylaxis vs no anaphylaxis), and number of food allergies (>3 food allergies vs < or = 3 food)	Internal consistency (test–retest)	Internal: correlations interitem. External: face, content, convergent/discriminative, construct	Yes	Flokstra-de Blok et al., The Netherlands [41]
Food allergy quality of life questionnaire-adult form spanish version (FAQLQ-AF)	29	Allergen avoidance and dietary restrictions; emotional impact; risk of accidental exposure; food allergy related health	≥18	Adults	≥3 foods = greater impact on QoL excellent internal consistency (Cronbach α, 0.95). S-FAQLQ-AF domains also had excellent internal consistency: α = 0.93 for allergen avoidance-dietary restrictions; α = 0.83 for emotional impact; α = 0.85 for risk of accidental exposure, and α = 0.66 for food allergy related health	Internal consistency (test–retest	Internal: correlations inter-items. External: face, content, convergent/discriminative, construct	Yes	Antolin-Amerigo et al., Spain [42]
Food allergy quality of life questionnaire-adult form swedish version (FAQLQ-AF)	29	Allergen avoidance and dietary restrictions; emotional impact; risk of accidental exposure; food allergy related health	≥18	Adults	O gender differences Allergen avoidance and Dietary Restrictions (AADR) highest score (lowest HRQL) number of food items to avoid did not influence QoL	Internal consistency (test–retest	Internal: correlations interitem. External: face, content, convergent/discriminative, construct	Yes	Jansson SA et al., Sweden [59]

**Table 3:** Factors with statistical significance that affect QoL in Fa

#	Factor	Article	Reference
1	Constant vigilance in the avoidance of specific foods to prevent an allergic reaction	Carrard et al.	[60]
2	Management of an acute reaction	Carrard et al.	[60]
3	Experience of anaphylaxis has a limited impact in QoL	Saleh-Langenberg et al.	[15, 36]
4	Allergies to fish and milk in adults and peanuts and soy in children caused greater HRQL impairment as compared to other foods	Saleh-Langenberg et al.	[15, 36]
5	Performing food challenge improved QoL irrespective of the outcome of the challenge (waines after 6 months in allergic patients)	Soller et al.	[49]
6	Perceived disease severity	Saleh-Langenberg et al.	[15, 36]
7	Country of origin	Saleh-Langenberg et al.	[15, 36]
8	Children >2 allergies	Sicherer et al.	[3]
9	Older children and those with mother or siblings affected by allergies	Wassenberg et al.	[17]
10	Oral induction of Tolerance (OIT) with peanut or cow milk: improves QoL	Factor JM et al., Carraro S et al.	[18, 19]

**Table 4:** QoL terminology [38, 41]

Concept	Definition	Concept	Definition
Reliability	Extent to which the questionnaire is repeatable and consistently produces the same results	Validity	Degree to which the questionnaire measures what it is intended to measure
Internal consistency	How well the items of a questionnaire relate to each other and to the total questionnaire. It is most commonly evaluated by Cronbach’s alpha. An alpha ≥0.70 indicates good internal consistency	Internal validity	Internal structure of the questionnaires and is usually evaluated by factor analysis, inter-items correlations and floor and ceiling effects
Test–retest	Reproducibility of the questionnaire over time. The questionnaire is completed on two occasions by the same patients in whom no change in the condition has taken place. It is most commonly evaluated by the intraclass correlation coefficient (ICC). An ICC ≥0.70 indicates good test–retest reliability	External validity	Relationship between the questionnaire and an external criterion (e.g. other measures of the same or different dimensions of health), and the most common types are face, content, convergent/discriminant and construct validity
		Face validity	Determined by expert opinion as to whether the questionnaire seems to measure HRQL related to the disease in question. Least rigurous form of validity. Type of external validity
		Content validity	Based on subjective assessment of the extent to which a questionnaire represents all dimensions of a construct. Type of external validity
		Convergent/discriminant validity	Assessed by calculating the correlation between the questionnaire and measures of similar or dissimilar constructs. Type of external validity
		Construct validity	Ascertained by calculating the correlation between the questionnaire and an independent measure, which reflects the severity of the disease in question. Type of external validity

Hitherto, strict avoidance of the culprit food(s) and the use of emergency medications are the pillars to manage this condition [3, 5]. Promising approaches such as specific oral or epicutaneous immunotherapy and the use of monoclonal antibodies are progressively being investigated worldwide. However, even that an increasing number of centers fulfill those approaches, they are not fully implemented enough in clinical practice.

The fact that neither the time of onset nor the intensity of the reaction is predictable can significantly influence QoL. Likewise, uncertainty when reading the ingredients and trace elements included in the food labelling on packaged food products may be bothersome for food allergic patients and their relatives [6]. The constant threat of exposure, need for vigilance and expectation of outcome can have a tremendous impact on their QoL [7, 8]. Several studies have analyzed the impact of FA on health-related quality of life (HRQL) in adults and children in different countries [7–11] (Tables 1, 2).

## Review

### Quality of life in children with food allergy

One of the most important issues about QoL in FA is to describe different predictors that shall contribute to modify HRQL. Identification of these predictors which have potential to decrease the patients’ HRQL could improve allergic patients by means of implementing adequate and specific approaches. [11] (Table 3).

In addition, we have to mention that a proven diagnosis of FA does not seem to be an independent predictor of HRQL, when compared to self-reported or perceived FA [11]. Although, HRQL in caregivers is heterogeneous and worse in those that are not followed-up at a FA referral clinic, in a tertiary center [12]. It has been stated that parents report a lower impact on HRQL than their allergic children (considering a similar perception of the allergy severity) [13]. In this line, it has been observed that, caregivers without food-allergic children may have different coping strategies than caregivers with FA children, revealing the importance of providing specific FA education to caregivers [14].

An elegant multicenter, multinational study describes several predictors of health-related QoL in European children [15]. Perceived disease severity, having a peanut or soy allergy, and the country of origin should be considered as contributors of the variance in HRQL (Table 3). Likewise, children with more than two food allergies had lower values of QoL scores compared with those with one or two food allergies [16]. Additionally, it has been observed that older children, the ones with severe systemic reactions, or those with mothers or siblings also affected by allergies, as well as girls, and children with multiple food allergies showed worse QoL scores [17].

Oral immunotherapy for different foods has been found to result in HRQL improvement, at least in participants with peanut or cow milk allergy [18, 19]. It has also been observed in a study comprising food-allergic children, where multiple-oral immunotherapies led to improvement in caregiver HRQL [20].

HRQL in food-allergic patients should be measured to have a global assessment of these patients, and for this reason specific questionnaires have been developed in recent times (Tables 1, 2), to be completed by parents [21, 22], but some of them also by children [23, 24]. These questionnaires should be short and easy to complete, to become both a useful and a suitable tool for evaluation of patients with food allergy.

One of the most used food allergy-related QoL questionnaires in children is probably the *Food Allergy Quality of Life Questionnaire (FAQLQ)*, which was developed and validated in Europe as a part of the EuroPrevall Project. These questionnaires include versions for children from 0 to 18 years old and for their parents [25]. But there are also other questionnaires that could be employed, for example the *Food Allergy Quality of Life*–*Parental Burden (FAQL*-*PB) Questionnaire* [26] developed in the US or the *Food Allergy Self*-*Efficacy scale for Parents (FASE*-*P)* that have been proved to be useful to identify areas where parents have less confidence in managing their child’s FA [22]. All these questionnaires have demonstrated good internal consistency (measured as Cronbach’s α), as well as good correlation with other generic and FA QoL questionnaires (Table 4).

It is important to perform lingual and cultural translations of existent questionnaires in order to ensure their suitability in a specific region or country, with its own socioeconomic reality and culture [27–29].

Briefly, for children there are general food questionnaire items that impair QoL to a greater extent, namely, “able to eat fewer products” and “always be alert as to what you are eating”, included in Allergen Avoidance and Dietary Restrictions domain; and the item “change of ingredients of a product” related with Risk of Accidental Exposure domain. The *FAQLQ*-*PF* showed that psychosocial impact in food-allergic children exerted a severe impact of on HRQL, due to the anxiety about food issues and the risk of a potential reaction [21] (Table 1).

### Quality of Life in Teenagers with Food Allergy

It is estimated that around 2 % of adolescents suffer from FA [30]. In healthy individuals the adolescence is a very critical time, characterized by accelerated growth and tremendous physiological, neurocognitive and emotional changes. In this context, chronic diseases like FA can have an even higher impact on the individual’s development and future wellbeing. Social isolation, depression, difficulties in school performance and leisure activities have been reported by food allergic adolescents as a result of their disease, along with the fear of allergic reactions [31–33]. On the other side, it is well known that a kind of incorrect belief of lack of risk leads teenagers to underestimate the severity of FA, as they think they will not die from any cause. It might result in risk-taking behaviours that can increase the risk of dying from FA [32, 34]. One of the major consequences is the reluctance to carry an epinephrine auto-injector, because the treatment is considered burdensome or simply not needed [35, 36]. According to recent data, the perceived burden of treatment is not directly associated with the overall HRQL, disease severity or trait anxiety, but it does significantly affect the non-compliance attitude towards epinephrine auto-injector and food restrictions [36]. Furthermore, a significant disagreement on health-related quality of life, mainly associated with adolescents’ rather than parents’ perceptions and characteristics, has been highlighted between parents and affected teenagers. Parents may not recognize the social impact of food restrictions or annoyance at having to carry self-injectable adrenaline [31, 37].

Up to now three tools for assessing HRQL in food allergic adolescents have been validated and can be used as reliable tool in daily clinical practice (Table 1).

It has been observed that UK and US teenagers, but not the Dutch ones, consider of primary importance the impact of FA on their social activities. US adolescents perceive their FA as a burden to others, but UK and Dutch teenagers do not confirm it. Dutch adolescents only experience the risk of accidental exposure as a concern. Support in managing FA is highly considered by UK teenagers but it does not appear to be the case for the Dutch and US ones [23, 38–40]. For these reasons the development of country-specific tools for assessing FA-related QoL should be one of the priorities in the FA management.

### Quality of life in adults with food allergy

Studies on food-allergic adult patients assessing QoL are scarce [41, 42] and the impact could be influenced by the fact that patients who have sought for medical help could have a worse QoL than those who have not actively looked for medical assessment [42, 43] (Table 2).

The *Food Allergy Quality of Life Questionnaire*-*Adult Form* (FAQLQ-AF) showed that uncertainty and anxiety seem to account for the greatest impact on HRQL in European food-allergic adults [7, 41, 42] (Table 2). Notwithstanding, both uncertainty and anxiety decreased in patients who underwent a double-blinded, placebo-controlled food challenge in the Netherlands [44].

The FAQLQ-AF is available for adults and was developed and validated in the context of the EuroPrevall Project, a multicenter European FA research project which objectives include analyzing the impact of food allergies on quality of life. It is currently available in several European languages [7–9, 41, 42] (Table 2).

**Fig. 1:**
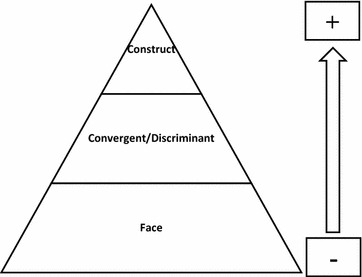
Different forms of external validity based on the rigor of the method of ascertainment

Construct validity of the FAQLQ-AF was assessed in patients from eight European countries, resulting as strong to very strong (Fig. 1). Moreover, internal consistency was excellent in all eight countries. A very interesting finding was that participants from eight European countries did not have comparable HRQL (as measured with total FAQLQ-AF scores). This result reinforces the value of the instrument, as it proves its sensitivity for differences in HRQL between populations with different socio-economic backgrounds [7] (Table 2).

In addition, studies have found significant differences in HRQL between countries, even when corrected for differences in perceived disease severity [15]. To unveil this aspect, *Saleh*-*Langenberg* et al. recruited a total of 648 European food-allergic patients (404 adults, 244 children) whom completed an age-specific questionnaire package including descriptive questions. Unexpectedly, the authors found that both for adults and children neither experiencing anaphylaxis nor being prescribed an epinephrine auto-injector (EAI) contributed to impairment of HRQL [15]. On the other hand, previous studies have shown that both confirmed and perceived FA impair equally HRQL [45].

The culture and traditions of eating might vary among different countries [42, 46], consequently, the impact of FA on quality of life shall diverge. Another important outcome was that forty-seven percent of all participants who reported anaphylaxis and who were diagnosed by a health care professional were not prescribed an epinephrine auto-injector, which corroborates previous findings about the suboptimal management of acute food-allergic reactions by both patients and physicians [47].

Other authors have suggested that as individual’s age, they probably become more aware of the severity of symptoms and may take into account the threatening effect of FA [42].

The healthcare cost in terms of FA has been investigated, in an elegant patient-based cost study. It has been reported that adults with ‘possible’ food allergy visited health professionals, on average, 11.17 (SD = 16.14) times per year compared with 7.11 (SD = 12.80) visits per year reported by controls. Similarly, children with ‘possible’ FA visited health professionals 10.75 times per year (SD = 13.23) compared with 6.56 (SD = 9.78) visits per year reported by controls. Consequently, food-allergic individuals had higher health care costs than controls. The mean annual cost of health care was international dollars (I$) 2016 for food-allergic adults and I$1089 for controls, a difference of I$927 (95 % confidence interval I$324–I$1530). A similar result was found for adults in each country, and for children, and interestingly, it was not sensitive to baseline demographic differences. Cost was significantly related to severity of illness in cases in nine countries [48].

In another study, QoL in adults with peanut allergy was compared with other disease groups. In contrast to children, the former group was observed to have better QoL than rheumatologic patients [45].

In addition, in a large population survey performed in Canada, individuals of low education and new Canadians self-reported fewer allergies, which may be due to genetics, environment, lack of appropriate health care, or lack of awareness of allergies, which could eventually reduce self-report [49].

Just to underline the impact that FA exerts in food-allergic patients, access to a 24-h telephone hotline specifically designed for this kind of patients in Ireland, significantly improved the measured QoL, and continued to do so for 6 months after the study time, even just two out of the 24 patients actually used it [50].

Moreover, some studies have shown the long-term positive effect food challenges yield on QoL. Unpredictably, this positive effect was not conditioned by the outcome of food challenges [51, 52].

## Conclusions

FA is suffered by patients but also by their relatives, friends and acquaintances [16, 26, 53] (Tables 1, 2). There have been described different factors that could modify HRQL in food allergic patients, considering as the most influential: perceived disease severity, age of the patient, peanut or soy allergy, country of origin and having allergy to two or more foods. Nevertheless, further studies are necessary to elucidate all these predictors and to achieve a good HRQL in food-allergic patients.

Over the last few years, several different specific QoL questionnaires for food-allergic patients have been developed and translated to different languages and cultures (Fig. 1). Tools designed to assess the impact of FA on HRQL should be always part of the diagnostic work up, in order to provide a complete basal assessment, to highlight target of intervention as well as to evaluate the effectiveness of interventions designed to cure FA [54–60]. HRQL may be the only meaningful outcome measure suitable and available for FA, measuring this continuous burden.
